# Electrical, Optical, and Anti-Microbial Behavior of Copper Nitrates-Doped Chitosan

**DOI:** 10.3390/nano16100601

**Published:** 2026-05-14

**Authors:** Ahmed A. Bhran, Abdelrahman G. Gadallah, Emad M. Ahmed, Azhar M. Elwan, Mohammed A. Farag, Mohamed M. M. Elnasharty

**Affiliations:** 1 Chemical Engineering Department, College of Engineering, Imam Mohammad Ibn Saud Islamic University (IMSIU), Riyadh 11432, Saudi Arabia; agadallah@imamu.edu.sa; 2 Department of Physics, College of Science, Taif University, P.O. Box 11099, Taif 21944, Saudi Arabia; makboul67@yahoo.com; 3 Department of Biochemistry, National Research Centre (NRC), 33 El Bohouth St., Dokki, Giza P.O. Box 12622, Egypt; z.mahmoud2006@gmail.com; 4 Physics Department, Faculty of Science, Al-Azhar University, Cairo P.O. Box 11884, Egypt; moha_far2010@yahoo.com; 5 Microwave Physics and Dielectrics Department, Physics Research Institute, National Research Centre (NRC), 33 El Bohouth St., Dokki, Giza P.O. Box 12622, Egypt; mohamed.elnasharty@googlemail.com

**Keywords:** chitosan, copper (II) nitrate, FTIR, optical analysis, dielectric properties of charge carriers, electrical impedance, antimicrobial activity

## Abstract

Chitosan-based copper composites have attracted considerable interest for biomedical and antimicrobial uses due to their biocompatibility, adjustable dielectric characteristics, and ion-mediated antimicrobial effectiveness. In this study, chitosan films doped with Cu(NO_3_)_2_, containing 3, 6, and 9 wt% of copper nitrate were produced using a solution-casting method at room temperature. This was done to explore the relationship between structural interactions, dielectric relaxation, optical properties, and antimicrobial efficacy. The resulting composite has been investigated physically using FTIR, XRD, optical analysis, and dielectric spectroscopy, and biologically for its antimicrobial activity. FTIR revealed the molecular structure of Cs-Cu(NO_3_)_2_ and changes resulting from new bond(s) formation and/or decomposition. XRD indicated that there are no peaks assigned for CuO, which weakens the composite antimicrobial activity. Optical analysis showed an increase in the band gap with copper (II) nitrate concentration over 3%. Additionally, the electrical impedance of the resulting composite increased by approximately one decade. A detailed electrical analysis of the charge-carrier types is provided. Moreover, the antimicrobial activity of chitosan is slightly enhanced by the additive copper (II) nitrate in a dose-dependent manner. The current research offers a mechanistic understanding of the structure–property relationships that govern the behavior of Cu(NO_3_)_2_–chitosan composites, emphasizing the significant influence of processing conditions on adapting of their dielectric and biological properties.

## 1. Introduction

Chitosan is attained by deacetylation of chitin. Chitin is the most abundant natural amino polysaccharide [1]. Chitosan was investigated in several studies as an antimicrobial material against a wide range of organisms, such as bacteria, yeasts, algae, and fungi, with different forms of chitosan, such as composites, solutions, and films. Therefore, chitosan is considered bactericidal or bacteriostatic, but it tends to be bacteriostatic rather than bactericidal. On the other hand, the chitosan activity against fungus is supposed to be fungistatic rather than fungicidal, making regulatory changes in both the host and fungus. The antifungal mechanism of chitosan depends on interfering directly with fungal growth, as observed in bacterial cells. Other studies showed that chitosan has a more rapid action on fungi than on bacteria [2,3,4,5].

Several studies used copper and copper alloys as antimicrobials on a wide band of Gram-positive bacteria, such as *Staphylococcus* spp., and *Enterococcus* spp., Gram-negative bacteria, such as *Campylobacter jejuni*, *E. coli*, *Acinetobacter* spp., and *Pseudomonas* spp., viruses like herpes simplex virus, influenza A virus, and human immunodeficiency virus, fungi and yeasts, such as *Candida* spp. They stated that copper nanoparticles are more effective on Gram-positive bacteria than Gram-negative ones [6,7,7,8].

Copper antibacterial activity is influenced by homeostasis mechanisms of copper in bacteria, humidity, strain specificity, adhesion, and manufacturing methods of antibacterial agents [9]. Copper has bactericidal action because it produces a hydroxyl radical, which contributes to several adverse reactions to cellular macromolecules, such as lipid and protein oxidation. The generated hydrogen peroxide causes increased production of toxic hydroxyl radicals [6,10].

A lot of research has been performed on the antibacterial properties of chitosan-copper composites. Mekahlia and Bouzid [11] investigated the antibacterial activity of seven chitosan–copper complexes with different copper concentrations. They concluded that the antibacterial activities of Chitosan-Cu(II) complexes are improved with increasing chelate ratios. Another study showed the positive influence of different composite materials based on chitosan, including chitosan with copper nanoparticles, which were prepared as thin films at room temperature [12]. Kadhim et al. [13] studied the antibacterial activity of copper oxide nanoparticles by preparing chitosan and copper nitrate (Cu_2_NO_3_) salt at 150 °C. Also, other studies showed the positive role of chitosan-copper composites against the Gram-positive and Gram-negative bacteria [14,15].

Despite the comprehensive research conducted on chitosan–copper composites, the connection between the incorporation of copper salts, dielectric relaxation behavior, charge transport mechanisms, and antimicrobial activity is still not fully understood, especially for composites created under mild processing conditions that maintain the original copper nitrate phase. Most prior research has concentrated on CuO nanoparticle systems generated at high temperatures, where thermal decomposition significantly modifies the chemical state of copper, thereby affecting the electrical and biological characteristics of the resulting composites [12,13,14,15]. In contrast, the behavior of Cu(NO_3_)_2_–chitosan systems cast at room temperature, where copper ions remain coordinated within the polymer matrix, has not been extensively studied [11].

Consequently, this study examines the structural, optical, dielectric, and antimicrobial characteristics of Cu(NO_3_)_2_-doped chitosan films that were produced using a low-temperature solution-casting technique. Special attention is given to the understanding of the electrostatic interactions between Cu^2+^ ions and the functional groups of chitosan, which play a crucial role in determining charge transport, dielectric relaxation, and ionic conduction mechanisms [16,17,18,19,20]. To establish correlations between structure and properties, FTIR, XRD, UV–Vis spectroscopy, and broadband dielectric spectroscopy were utilized, while the antimicrobial efficacy was assessed against selected Gram-positive bacteria, Gram-negative bacteria, yeast, and fungi [6,7,7,8,9,10,11,12,13,14,15]. The objective of this research is to offer mechanistic insights into how the incorporation of copper nitrate alters the physicochemical properties of chitosan and to elucidate the impact of processing conditions on the multifunctional performance of the resultant composite films.

## 2. Materials and Methods

### 2.1. Sample Preparation

Chitosan (C_6_H_11_NO_4_)_n_, with molecular weight of 100–300 kDa, was bought from Acros Organics (Geel, Belgium). Chitosan was dissolved in distilled water containing 2% acetic acid. The obtained mixture was stirred for 3 h at room temperature. Then, Copper nitrates were added in 3, 6, and 9 wt% with continued stirring. The copper nitrates and acetic acid were purchased from Sigma-Aldrich (St. Louis, MO, USA). The solutions were then sonicated to ensure homogeneous dispersion of the copper nitrate. Finally, the mixtures were poured into Petri dishes and dried at 40 °C for 24 h [20].

As shown in Table 1, 0.5 g of chitosan was dissolved in 100 mL of acidified water (2% acetic acid). The amounts of Cu(NO_3_)_2_ added were as follows: 15 mg for 3 wt%, 30 mg for 6 wt%, and 45 mg for 9 wt%. The molecular weight of Cu(NO_3_)_2_ is 187.55 g/mol, corresponding to a copper content of 33.88% (0.3388) by weight in copper(II) nitrate.

### 2.2. FTIR Spectroscopy

FTIR analysis was done using a Bruker optics VERTEX 7000 Fourier Transform Infrared Spectrometer (Bruker Optics GmbH, Ettlingen, Germany), having a spectral span of 4000–400 cm^−1^ was used.

### 2.3. XRD Characterization

XRD analysis was carried out using an X-ray diffractometer (XRD), a Rigaku SmartLab, HyPix-400 instrument (Rigaku Corporation, Tokyo, Japan) with CuKα radiation (λ = 1.5406 Å) over a 2θ range of 5–80° (40 kV, 50 mA).

### 2.4. Optical Analysis

The UV–vis absorption spectrum was recorded by a UV–Vis spectrophotometer (JASCO V-630, Tokyo, Japan) using a 1 cm path length quartz cell.

### 2.5. Dielectric Spectroscopy

Dielectric measurements were performed on chitosan doped with copper nitrate using a Broadband dielectric spectrometer, Concept 40, from Novo Control, Montabaur, Germany. The samples were measured using two brass electrodes, the diameter of the small one was 10 mm, and placed within the alpha impedance analyzer’s cell, the measuring device of Concept 40, measuring cell. The frequency range investigated was from 0.1 Hz to 20 MHz, applied voltage was adjusted to 1 V_rms_, and the temperature range from (10–40 °C), was controlled by the Concept 40 system, within the measuring cabinet using N_2_ gas under vacuum with a temperature accuracy of 0.1 °C. The calculations have been carried out by the formula:

#### 2.5.1. Electrical Impedance

Equation (1) is used to calculate the electrical impedance of the material when an AC voltage is applied.

(1) $$Z^{*}\left(\omega ,T\right)=\frac{R_{DC}(T)}{{\left(1+{\left(i\omega \tau \;\left(T\right)\right)}^{\alpha }\right)}^{\beta }}$$

where Z* is the complex impedance, R_DC_ is the direct current resistance, i equals $\sqrt{-1}$, ω is the angular frequency (2πν), T is the temperature, and τ is the relaxation time. The α and β shape parameters are defined as 0 < α ≤ 1 and 0 < β ≤ 1.

#### 2.5.2. Dc Conductivity

Equation (2) (Arrhenius equation) describes how electrical conductivity changes with temperature.

(2) $$\sigma =\sigma_{o}\;exp[-\frac{E_{a}}{RT}]$$

where σ is conductivity at absolute temperature T, σ_o_ is a pre-exponential factor, *E_a_* is the activation energy and R is the gas constant.

#### 2.5.3. Complex Modulus

Equation (3) describes the electric modulus, which is another way of studying dielectric relaxation.

(3) $$M^{*}\left(\omega \right)=\varepsilon_{s}^{-1}-\frac{\varepsilon_{s}^{-1}}{{\left(1+{{(i\omega \tau }_{\sigma })}^{\alpha }\right)}^{\beta }}$$

where M* is a complex electric modulus, ε_s_ is the static dielectric constant, *α* and *β* are shape parameters, *τ_σ_* is the dc conductivity relaxation time [20,21,22,23].

### 2.6. Anti-Microbial Activity

The antimicrobial activity of chitosan doped with copper nitrate in concentrations 3, 6, and 9% was investigated by the agar disc diffusion method. Four different test microbes namely: *Staphylococcus aureus* (G+ve), *Escherichia coli* (G−ve), *Candida albicans* (yeast), and *Aspergillus niger* (fungus) were used. Nutrient agar plates were heavily seeded uniformly with 0.1 mL of 10^5^–10^6^ cells/mL in case of bacteria and yeast. A Czapek-Dox agar plate seeded by 0.1 mL the fungal inoculum was used to evaluate the antifungal activities. Films (1 cm) were placed on the surface of inoculated plates. Then plates were kept at low temperature (4 °C) for 2–4 h to allow maximum diffusion. The plates were then incubated at 37 °C for 24 h for bacteria and at 30 °C for 48 h in upright position to allow maximum growth of the organisms. The antimicrobial activity of the test agent was determined by measuring the diameter of zone of inhibition expressed in millimeters (mm). The experiment was carried out more than once and mean of reading was recorded [24].

## 3. Results and Discussion

### 3.1. FTIR Spectroscopy of Chitosan-Copper Nitrate Composite

**Figure 1:**
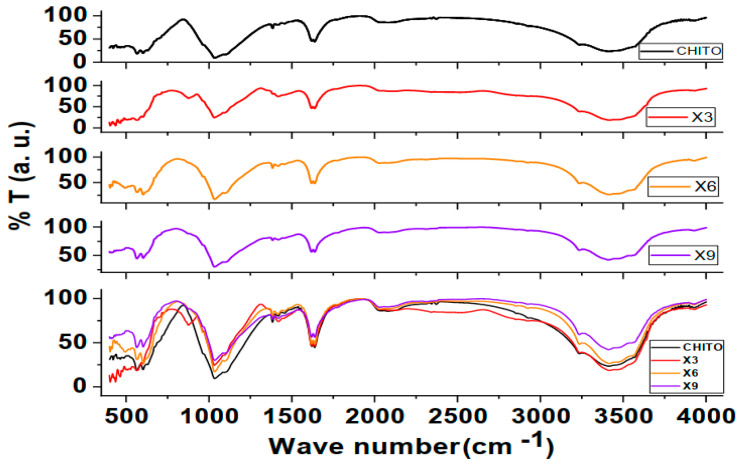
FTIR of chitosan, upper, and chitosan films with different copper nitrate concentrations, 3, 6, and 9% resp.

It can be seen from Figure 1 that the copper nitrate is well incorporated within the chitosan film. At 3400–3300 cm^−1^, primary and secondary amine bonds, N-H, along with O–H stretch, are located in this region. There are small C-N peaks at about 2340–2375 cm^−1^. The C=O bond can be located at about 1640 cm^−1^, C-O at about 1200 cm^−1^. C-O-C found at about 1050 cm^−1^. The N–O stretching asymmetric and symmetric vibrations occur around 1380–1480 cm^−1^. Also, the presence of N-O stretching within NO_3_ is found only in X3 sample and apparently it is from the nitrates which were not completely reacted with the chitosan. This peak disappeared in other samples containing higher concentrations of CuNO_3_, indicating complete miscibility. It is noticed that increasing the ratio of copper nitrate within the chitosan film resulted in a general decrease in all peaks except from 1200–1600 cm^−1^ [25,26,27,28,29].

Mechanistic comment on FTIR Spectroscopy: Starting with O-H/N-H stretching, in Figure 1, observed near 3430 cm^−1^, the peak shifts slightly by 0, 25, and 50 cm^−1^ for 3, 6, and 9% Cu(NO_3_)_2_ additions, respectively. This slight red shift indicates Cu^+2^ adsorption, weakening O-H/N-H bonds, and increases with Cu(NO_3_)_2_ content. For C-H stretching, the peak position changes were negligible, indicating no degradation of the chitosan backbone. The amide I C=O at 1650 cm^−1^ decreased by 0, 4, and 9 cm^−1^ for the three concentrations, respectively. This amide peak undergoes a slight shift during coordination with Cu. The NH_2_ bending peak at about 1560 cm^−1^ red-shifts by 0, 7, and 15 cm^−1^, and this peak shows the strongest shift, indicating Cu-N bonding. The C–O–C glycosidic region at about 1060 cm^−1^ red-shifts by 0, 7, and 13 cm^−1^. This shift indicates coordination of Cu with the chitosan backbone. At low wavenumbers around 500–600 cm^−1^, an emerging peak is very weak for the 3%, evolves in the 6%, and becomes clearer in the 9% sample. This peak indicates Cu-O at about 600 cm^−1^ and Cu-N at 500–600 cm^−1^.

From the provided data, it is concluded that bands for the following functional groups indicate: NH_2_ is a primary binding site; OH is associated with hydrogen-bond disruption; C=O shows minor coordination, as there is no change at 2920 cm^−1^, which points out that the backbone is intact; and the new bonds (Cu-O/Cu-N) appear at high concentration. The absence of this peak points out that there are no CuO nanoparticles in the composite. Another very important point is the effect of asymmetric stretching of free nitrate ions, NO_3_, which should appear as a sharp peak about 1350–1380 cm^−1^, and it becomes broader (1300–1500 cm^−1^) if such ions are coordinated. However, this peak is missing from the FTIR spectra. This leads to the conclusion that the nitrate ions are not free. This is because preparation, casting, and drying of samples were performed at room temperature. Also, there is no peak for CuO in the FTIR spectra, which should appear at 480–650 cm^−1^, a strong monoclinic phase of Cu-O stretching peak [13]. In other words, they’re still bound to the Cu atoms. Consequently, all the former interactions are purely physical and not chemical. That is why the backbone of chitosan is intact despite NH_2_ being a primary interaction site. It also explains the decrease in conductivity after the addition of Cu(NO_3_)_2_. The result is physical charge attraction among the lone pairs on the main atoms within the functional groups, such as NH_2_, C=O, and OH, on one side, and the Cu atom of Cu(NO_3_)_2_ on the other. This restricts the movement of charges further and explains the weak antibacterial effect, which will be presented later.

### 3.2. XRD Analysis

The XRD patterns of the Cu(II) nitrate–doped chitosan films show a doping-concentration-dependent structural development that can be explicated within the basis of polymer–salt interactions [30,31].

**Figure 2:**
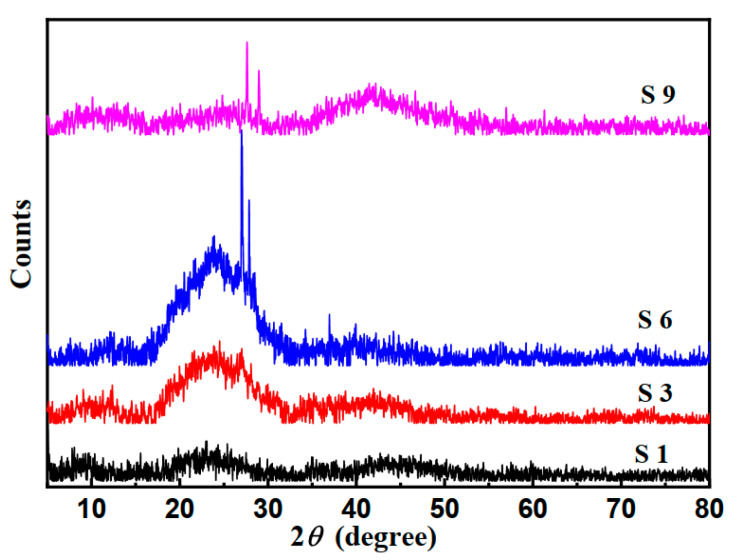
XRD patterns of chitosan films doped with Cu(II) nitrate at (S1) 0%, (S3) 3%, (S6) 6%, and (S9) 9% wt% concentration, recorded using Cu Kα radiation (λ = 1.5406 Å) over a 2θ range of 5–80°.

As presented in Figure 2, Humps at ~10° and ~20° specify characteristic chitosan reflections. Sharp spikes near 2θ ≈ 27° indicate reflections referred to a crystalline Cu(NO_3_)_2_·3H_2_O phase. The progressive increase in peak sharpness and intensity from S3 to S6 evidences increased crystallinity of the dopant phase. The partial broadening observed at S9 relative to S6 suggests that the salt starts to interrupt the chitosan matrix at higher doping levels. In the undoped chitosan film (S1), the diffraction profile is characteristic of a semi-crystalline biopolymer. The two broad humps at ~10° and ~20° are signs of the chitosan orthorhombic unit cell, as found in Saito et al. [32] who assigned them to the (020) and (110) reflections, respectively. The FWHM values (2.5° and 4.0°) and low crystallinity index (~23%) are consistent with a film cast from acetic acid, where solvent–polymer interactions partially disrupt the interchain hydrogen-bonding that drives crystallinity in solid-state chitosan [33].

Upon introduction of 3 wt% Cu(NO_3_)_2_ (S3), weak but prominent new reflections emerge at 22.6° and 26.4°. Their d-spacings (3.93 and 3.37 Å) match published lattice spacings for Cu(NO_3_)_2_·3H_2_O (ICDD card 00-015-0014). The Scherrer domain sizes derived from these peaks (54 and 82 nm) are significantly larger than what would be predictable for purely dissolved or amorphously dissipated Cu^2+^ ions, providing direct structural indication for nanoscale Cu salt crystallite formation at 3% loading. The concordant small increase in CI (to 26%) suggests that Cu^2+^ coordination with chitosan’s –NH_2_ groups, forming stable Cu–N bonds [34], may locally promote chain alignment by reducing chain mobility.

At 6% doping (S6), the appearance of the intense 27.0° reflection (I ≈ 439 counts, D ≈ 91 nm) marks a qualitative change in structural character. The sharpness and intensity of this reflection, together with the supporting reflections at 22.9° and 32.0°, show that at this concentration the Cu(NO_3_)_2_·3H_2_O phase has overlapped a percolation or nucleation threshold, forming extended, well-ordered crystalline domains within the chitosan matrix. Such behavior has been described as ‘crystallization-induced phase segregation’ in polymer–salt composites [35]. The concomitant peak at 32°, with a d-spacing of 2.79 Å, partially overlaps with the (110) reflection of CuO (d = 2.75 Å, ICDD 00-041-0254), proposing trace oxidation. Given that the samples were prepared under ambient conditions, minor oxidation of Cu(II) nitrate to CuO during casting or drying cannot be preclude.

At 9% loading (S9), the reduced crystallite size (74 nm at 27.6°) and decreased peak intensities despite increased Cu content signify strong evidence of cooperative disruption. The Cu^2+^ ions, more than the saturation capacity of chitosan’s chelation sites, interact with the already-formed Cu(NO_3_)_2_·3H_2_O crystallites, introducing lattice defects and reducing domain coherence. A parallel mechanism is the plasticization of the chitosan matrix itself: excess NO_3_- anions from the salt may contest with the inter-chain hydrogen bonds, increasing free volume and reducing the ordered chitosan domain size. This clarification is consistent with dielectric and mechanical softening reported for chitosan–salt films at high ionic concentrations [36,37].

The mean crystalline domain size (D) is calculated using Scherrer equation (Equation (4)) for sharp reflections as presented in Table 2. The results in this table are the upper-bound estimates, as the instrumental broadening was not subtracted.

(4) D=kλ /(β cosθ)

where *k* = 0.9 (dimensionless shape factor for spherical crystallites), λ = 1.5406 Å (Cu Kα wavelength), β = FWHM in radians, and θ = Bragg angle in radians. FWHM values were estimated from the peak profile width. It should be noted that the instrumental broadening was not subtracted.

Table 3 illustrates the details of all peaks found in the studied samples and assigns them to their origins in the samples. Where, D is the crystallite size estimated by Scherrer equation using K = 0.9, and λ = 1.5406 Å. FWHM values estimated from the peak profile fitting. It is noticed that CuO is only found as a trace mixed phase in sample 6. The absence of CuO explains the weak antimicrobial effect of the samples as compared to other work in the literature. The results of Table 3 show that all found phases are attributed either to chitosan and/or Cu(NO_3_)_2_·3H_2_O salt.

The ratio of the net area under crystalline peaks to the total diffraction area is known as the crystallinity index (CI) as listed in Table 4. Using the peak to background intensity ratio near 20° chitosan as a reference, CI was valued and tabulated in Table 4 linking them to their origin.

Observed crystallinity is due to Cu(NO_3_)_2_·3H_2_O, not CuO as would be thought usually, and this is due to the preparation and casting at low temperatures. The highest crystallinity was reached at 6 wt% sample, and crystallinity disappeared again at 9 wt% sample. This is evidence of polymer matrix disruption, as observed in salt systems doped poly vinyl alcohol [38]. Taken together, the XRD data establish a clear structure–property map as described below:

(i) At low doping (3%), Cu(II) is primarily ionically related to the chitosan matrix with incipient nano-crystallite formation;
(ii) At intermediate doping (6%), a distinct, well-crystallized Cu(NO_3_)_2_·3H_2_O micro-phase coexists with an ordered chitosan matrix;
(iii) At high doping (9%), excess Cu(II) salt disrupts both its own crystalline order and the chitosan matrix, yielding a more homogeneously disordered composite.

This non-monotonic crystallinity–concentration relationship has important implications for the design of Cu-doped chitosan materials, suggesting that 6% characterizes an optimum dopant loading for applications requiring well-defined crystalline Cu phases, while concentrations above this threshold should be avoided if structural integrity is paramount. These data indicate that Cu(II) nitrate not only did not disintegrate within the chitosan matrix, but also weakened its electrical positivity by diffusing within the matrix and physically bonding to its positive and negative functional groups, electrostatically forming Cu-N; Cu-O and the nitrate groups did the same with partially positive atoms like H, using its O atoms.

### 3.3. Optical Analysis of Chitosan-Copper Nitrate Composite

**Figure 3:**
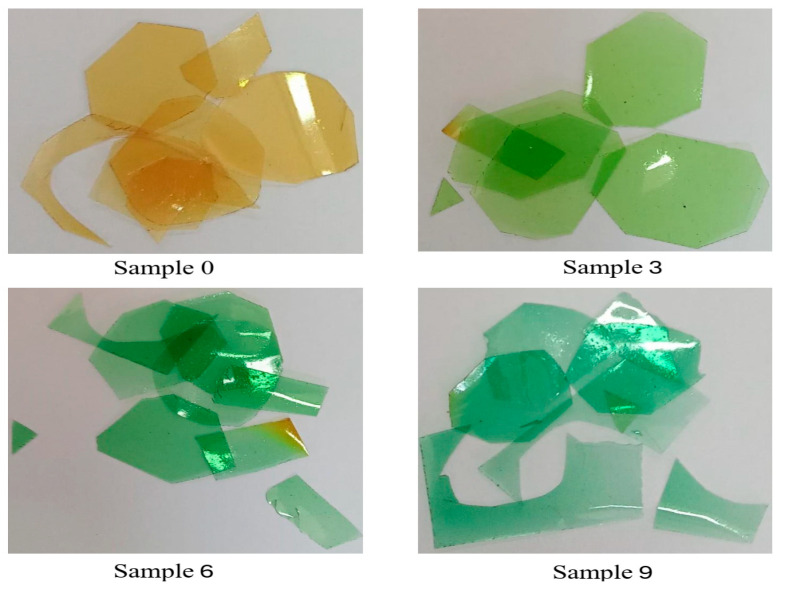
Optical appearance of the samples.

Optical properties, such as band gap energy and refractive index, are among the most important physical properties for films. From Figure 3, the blank sample (Sample 0) is transparent with a yellowish color. By adding copper nitrate (Samples 3, 6, and 9), the samples earned a green color. This appearance is due to the Cu ion optical absorption. The samples show no accumulation of copper in the prepared samples which indicates the well distribution of the copper inside the polymer.

**Figure 4:**
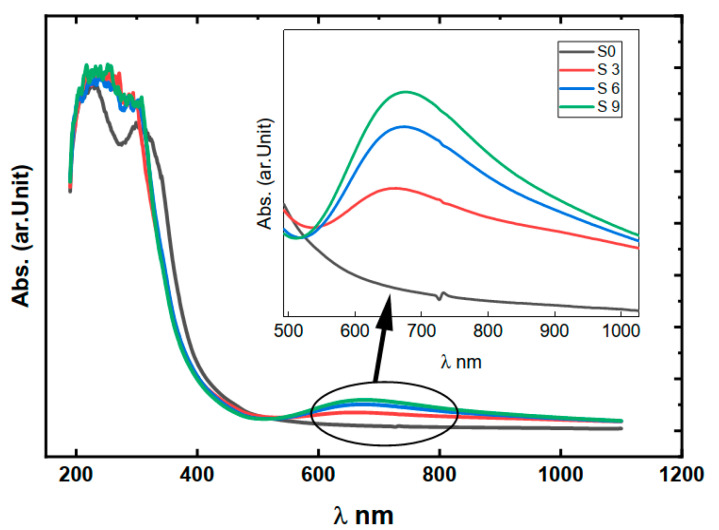
Optical absorption of the prepared samples.

Figure 4 shows the optical absorbance of the prepared polymer without the addition of copper nitrate shows no absorption peaks with a wide transparency range followed by an abrupt increase in absorption near the optical band edge.

**Figure 5:**
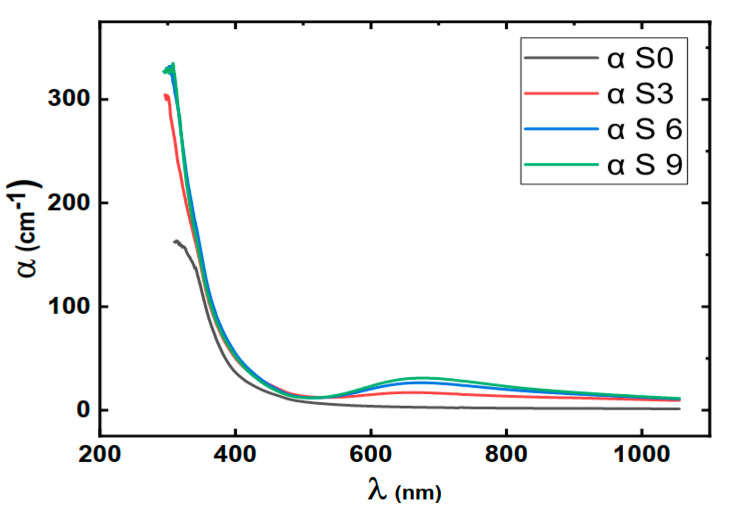
Optical absorption coefficient (*α*) of the prepared samples.

As displayed in Figure 4, after the addition of copper nitrate, the absorption peak centered around 650 nm, starts to appear. This band could be due to the presence of Cu^+2^ in the samples [39]. The absorption peak intensity increases by increasing the copper nitrate concentration in the samples. This confirms that the Cu ion is the origin of the sample’s colors and absorption. Optical absorption coefficient (*α*) could be calculated by Equation (5).

(5) $$\alpha =2.3\;\frac{A}{d}$$

where A is the optical absorbance and d is the sample’s thickness [40]. The absorption coefficient of the samples is shown in Figure 5.

Figure 5 shows that the absorption coefficient of the samples increases with the increase in the copper nitrate content. This again confirms that the optical absorption is due to the insertion of Cu ions into the polymer’s chains.

**Figure 6:**
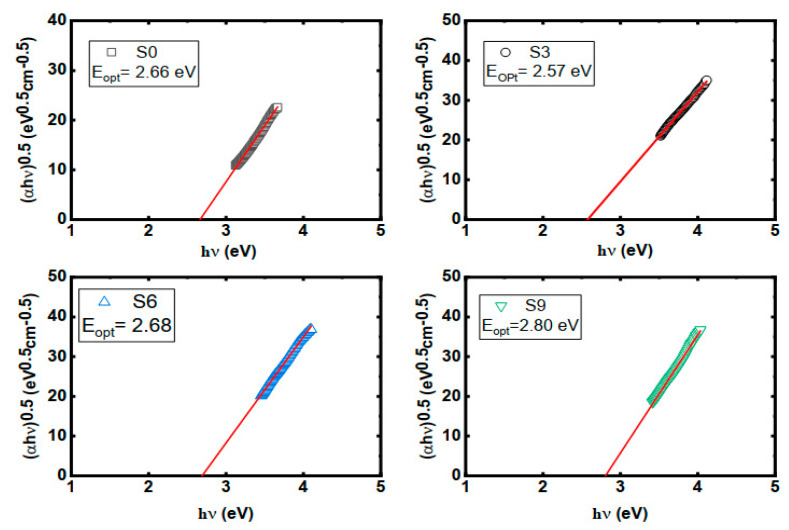
Touc’s relation of the prepared samples.

Optical band gap (Eopt) could be determined by extrapolation of the linear part of Mott-Davis relation [41] as shown by Equation (6).

(6) $$\alpha \left(h\upsilon \right)=B{\left(h\upsilon -E_{opt}\right)}^{n}$$

where, h is Planck’s constant, *υ* is the frequency of the optical ray, B is a constant and (n) is the index that has different values (2, 3, 1/2, 1/3). The value of (n) is determined according to the type of materials [42]. In this work n = 2 for the amorphous prepared polymer. To determine the energy gap (Eopt) according to Touc’s relation, the variation of (αhυ)1/2 vs. (hυ) was plotted as shown in Figure 6.

Figure 6 shows that the bandgap decreases with the increase in copper nitrate up to 3%. By increasing the copper nitrate over 3% the band gap increases. By calculating the bandgap, refractive index (n) could be calculated according to the Equation (7) [43].

(7) $$\frac{n^{2}-1}{n^{2}+2}=1-\sqrt{\frac{E_{g}}{20}}$$

The obtained band gap and refractive index results are shown in Table 5. All samples show a relatively high refractive index decreasing with the increase in copper ions content inside the polymer.

### 3.4. Dielectric Analysis of Chitosan-Copper Nitrate Composite

**Figure 7:**
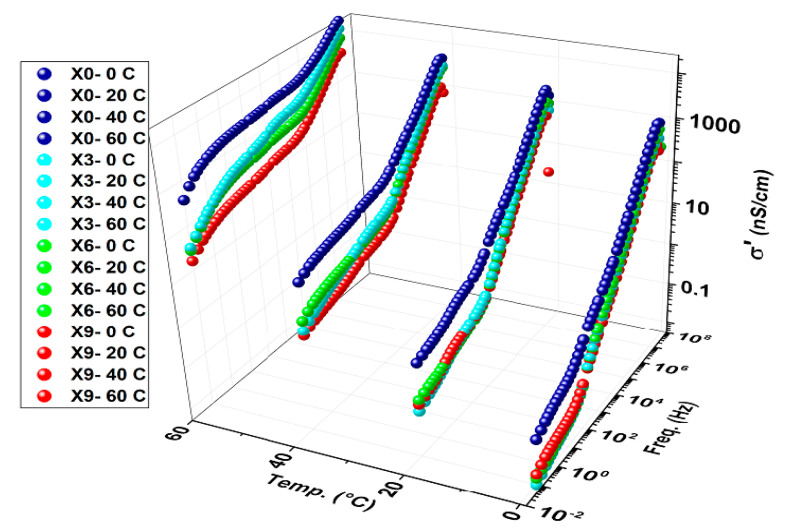
3D representation of real conductivity of chitosan and chitosan-copper nitrate composite (3%, 6%, 9%) scanned along frequency within the temperature window.

Real conductivity (see Figure 7) shows that the conductivity of the chitosan film decreases with the addition of copper nitrates, and the decrement continues with rising copper nitrates concentration. This implies that Cu(NO_3_)_2_ is trapped within the chitosan matrix in its nano form or interacts with the chitosan bonds with similarly existing bond types, as there are no indications in the FTIR spectra to inform about new bond formation, as shown in Figure 1. In fact, the absence of both peaks representing CuO NPs and free nitrate groups 480–650 and 1380 cm^−1^, respectively, along with the raise of band gap with Cu(NO_3_)_2_ concentration explains the decrement of conductivity. Direct proportionality between conductivity and temperature indicates a semiconductor behavior of the composite film formed.

**Figure 8:**
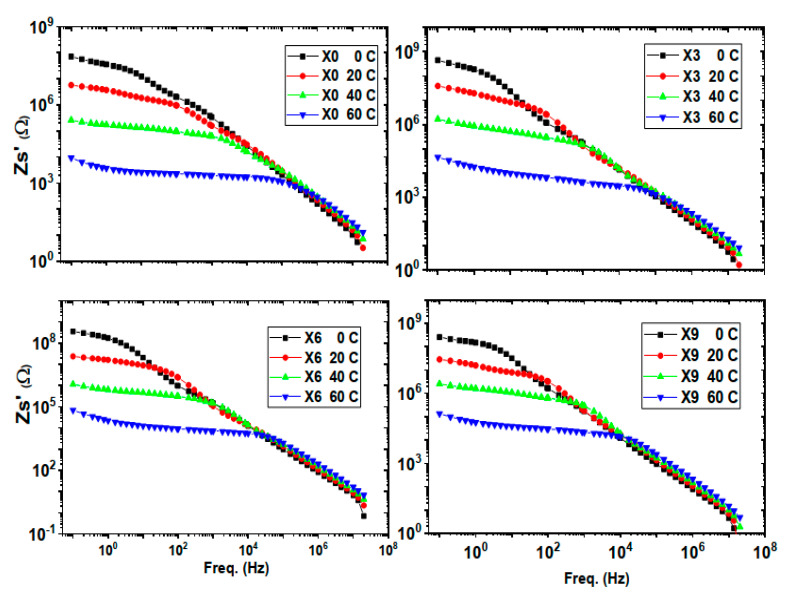
Real impedance of chitosan and chitosan-copper nitrate composite with frequency at different temperatures.

Real impedance, Figure 8, decreases with temperature increment, extending stability frequency independent plateau expressing semiconductor attitude of the composite. Changing Cu(NO_3_)_2_ concentrations using the current ratios provided an increment of about one decade of the real impedance. This represents a semiconductor behavior of the composite.

**Figure 9:**
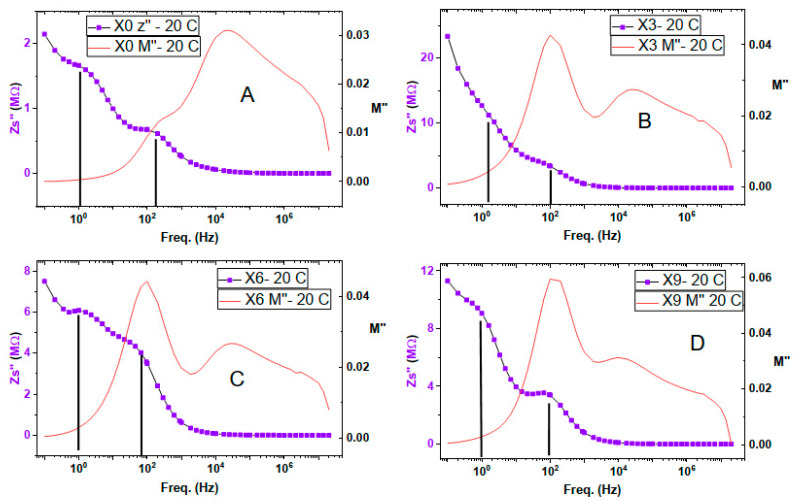
Imaginary part of impedance, left axes, and imaginary part of electric modulus, right axes, spectra of composite samples at 20 °C. (**A**): X0, Chitosan, (**B**): X3, 3% Cu(II) nitrate doped Chitosan, (**C**): X6, 6% Cu(II) nitrate doped Chitosan, and (**D**): X9, 9% Cu(II) nitrate doped Chitosan.

Figure 9 displays two relaxation peaks in the imaginary part of impedance in the low-frequency range. Imaginary impedance peaks represent capacitive impedance, which means that we have two different ionic conduction mechanisms, having two different speeds, one for each type of charge carrier.

**Figure 10:**
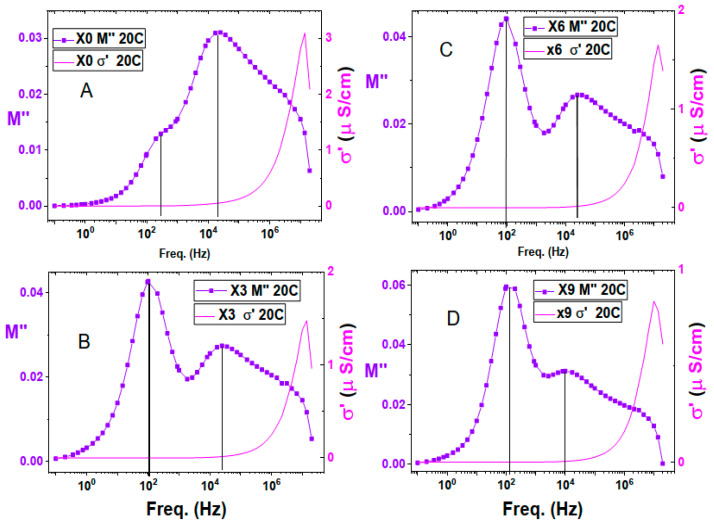
Imaginary part of electric modulus, M”, and real conductivity versus frequency at 20 C. (**A**): X0, Chitosan, (**B**): X3, 3% Cu(II) nitrate doped Chitosan, (**C**): X6, 6% Cu(II) nitrate doped Chitosan, and (**D**): X9, 9% Cu(II) nitrate doped Chitosan.

By combining the electric modulus, M”, with the impedance, Z” in Figure 9, we can find that the first relaxation peak at a very low frequency, about 1 Hz, does not appear in the modulus relaxation curve. The real part of the electric modulus and the real conductivity shown in Figure 10 display that this frequency range is dominated by electrode polarization as found in the real conductivity spectrum. This range is damped in the real part of the electric modulus curve, which is known to dampen electrode polarization. In addition, the Cole-Cole plot of conductivity shows two conductivity mechanisms below 10 kHz. This information led to the conclusion that the 1st relaxation peak, at about one Hz of the imaginary part of the impedance, is a result of electrode polarization.

The 2nd capacitive impedance relaxation peak occurs at about 100 Hz and coexists in both the imaginary impedance and imaginary electric modulus spectra, Figure 8. This indicates that it is a long-range charge hopping of the grain boundaries [44].

Chitosan film displays three relaxation processes in the electric modulus spectrum, Figure 10. The one at high frequency, about 3 MHz, and the intermediate one at about 20 kHz have no counterparts in the imaginary impedance curve in Figure 10A. Which means they represent the short-range local hopping of grain polarization. The impact of copper nitrate addition on the intermediate relaxation peak led to a slight reduction of its strength at 3 and 6% concentrations, then its strength was restored at 9% concentration of copper nitrate. As for its location, it was right-shifted at 3 and 6% then left-shifted at 9% concentration to reach a peak position at about 10 kHz. The existence of these two relaxation peaks within the DC conductivity range of the real conductivity means that they represent two different ionic motions with different speeds.

The third relaxation situated in the lower frequency is around 250 Hz, Figure 10A, and has the lowest strength of the three relaxation peaks of chitosan. Incorporation of Cu(NO_3_)_2_ with chitosan has a great influence on this relaxation peak, where it enhanced its strength to become the highest strength among all chitosan peaks, Figure 10D. In addition, it causes a delay in the relaxation time to about double the time of Figure 10A, and the peak maximum is located at 100 Hz. This relaxation is represented in the imaginary impedance curve illustrated by Figure 9. This means that this relaxation is of long-range ionic conduction type and performed by free-charge carriers.

**Figure 11:**
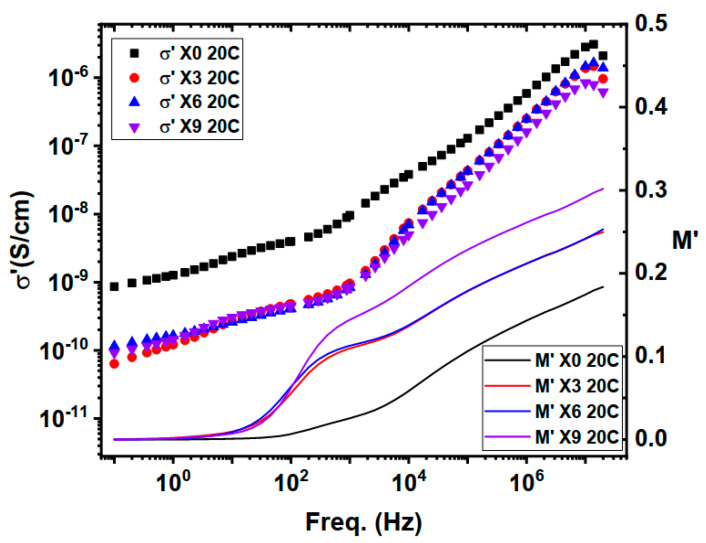
Plot of the real part of conductivity, left axis, and the real part of electric modulus, right axis, along the scanned frequency window.

In Figure 11, attention is focused on the lower frequency part of the real conductivity and modulus spectrum, where the real conductivity exhibits electrode polarization. This is confirmed by the damped behavior of the real electric modulus in the same frequency range, indicating the occurrence of electrode polarization.

### 3.5. Antimicrobial Activity

**Figure 12:**
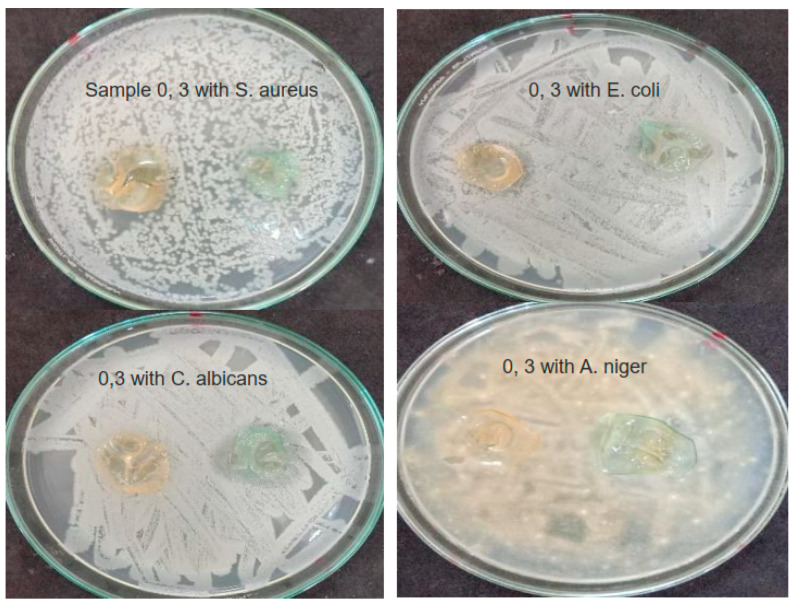
Antimicrobial activity of chitosan and chitosan film with copper nitrate concentration (3%).

**Figure 13:**
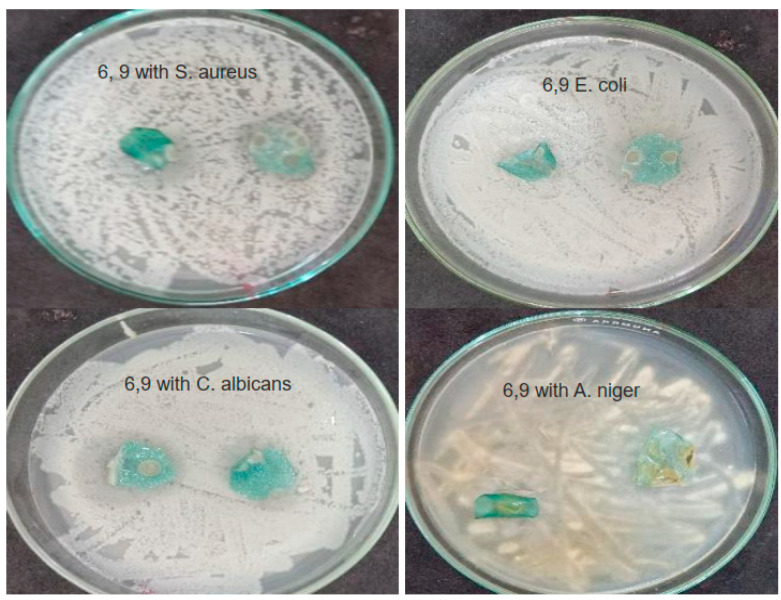
Antimicrobial activity of chitosan and chitosan films with copper nitrate concentrations (6, 9%).

The results of antimicrobial activities of chitosan and chitosan films with copper nitrate concentrations (0, 3, 6, and 9%) against *Staphylococcus aureus* (G+ve), *Escherichia coli* (G−ve) *Candida albicans* (yeast) and *Aspergillus niger* (fungus) are shown in Figure 12 and Figure 13 and Table 6.

From the results, it is evident that chitosan, Cs, exhibits no antimicrobial activity against all types of microbes, and none of the samples showed antimicrobial activity against *Escherichia coli*. Cs-Cu(NO_3_)_2_ (3%) had antimicrobial effects against *Candida albicans* and *Aspergillus niger*. While Cs-Cu(NO_3_)_2_ (6%) had antimicrobial activities against *Staphylococcus aureus* and *Candida albicans*, stronger than the lower concentration (3%). Cs-Cu(NO_3_)_2_ (9%) had more antimicrobial activities on *Staphylococcus aureus*, *Candida albicans*, and *Aspergillus stronger* than lower concentrations (3 and 6%).

The possible reason for no remarked antimicrobial activities is that chitosan was in a film form that, in many cases, folded and did not preserve its contact area intact with the culture media in the petri dish. This, as a consequence, decreased the contact area, where the diffusion of the free ions occurs. On the other hand, the additive copper (II) nitrate could induce the antimicrobial effect of the samples, and this effect was more pronounced at higher concentrations. High concentrations of Cu(II) can cause a toxic effect on the microorganisms, such as enzyme inactivation, blocking the protein’s functional groups, production of free radicals by membrane-bound copper, and changing membrane integrity [11,45].

Despite the enhancement of chitosan’s antimicrobial activity after adding Cu(NO_3_)_2_, the results were lower than expected. The interpretation of these results is attributed to the confinement of charge carriers to short-range, local polarization, and the retention of the copper atoms within chemical and electrostatic bonds formed among the nitrogen atoms and the hydroxyl groups in the chitosan and Cu(NO_3_)_2_ molecule, in addition to the physical bonds among the amine groups and the oxygen atoms of the nitrate groups. Such bonds limit the copper conduction band’s electrons from moving freely. Based on FTIR investigation, the absence of CuO NPs and free nitrate ions peaks indicates the complete miscibility of Cu(NO_3_)_2_ as intact molecules within the chitosan. Also, XRD patterns for CS-Cu(NO_3_)_2_ samples show no CuO peaks as well. This points to electrostatic physical bonding with the chitosan structure, which limits the copper atoms’ capability as an antimicrobial agent and, at the same time, decreases the total positive charge characterizing chitosan, attenuating its ability to electrically attach bacteria. That is why the composite has no antibacterial effect on the Gram-negative bacteria, E.coli, which has a more resistant cell membrane than the Gram-positive ones.

Furthermore, the band gap value of CuO NPs is 1.8 eV, showing a high ZOI 40 mm [13]. The band gap is much larger, leading to difficulty in ionic motion. As a result, both conductivity and antimicrobial inhibition zones had lower values.

Based on the dielectric data, the limited increase in anti-microbial activity of Cs-Cu(NO_3_)_2_ is due to the long-range dc ionic conduction, occurring at about 100 Hz. In the current composite, we have three modulus relaxations, only one shows a long-range ionic conduction at about 100 Hz. In addition, the impedance spectrum has two relaxations, only one of which represents a long-range ionic conduction at the same low frequency. This interpretation clarifies that the antimicrobial effect relies on the long-range charge carriers, which are least expected in this composite due to drying conditions at room temperature and are not at higher temperatures, that can cause evaporation of nitrate groups and leave the copper ions free within the composite.

## 4. Conclusions

In this work, chitosan composite films doped with Cu(NO_3_)_2_ were successfully produced using a low-temperature solution-casting method, which facilitated the retention of the copper nitrate phase and enhanced strong electrostatic interactions within the polymer matrix. Incorporating copper nitrate with chitosan led to its diffusion within the chitosan matrix. Drying the solution of the composite freely without heating contributed to protecting the copper nitrate from heat decomposition, allowing it to form physical and chemical bonds with different functional groups of the chitosan. Increasing the ratio of copper nitrate within the chitosan film resulted in a general decrease in all peaks of the FTIR spectrum, except for the range of 1200–1600 cm^−1^. The absence of both peaks, representing CuO and nitrate ions, shows the complete miscibility of Cu(NO_3_)_2_ as intact molecules within the chitosan. This points out its electrostatic physical bonding with the chitosan structure. These data were confirmed by XRD pattern, which clearly indicate that the only found peaks are assigned to either chitosan and/or Cu(II) nitrate salt. From the optical analysis, an increase in the band gap was observed after the copper nitrate concentration exceeded 3% formed an obstacle against ionic motion, lowering both conductivity and antimicrobial effects. Additionally, the newly formed composite of chitosan exhibits increased electrical impedance and a semiconductor-like behavior with respect to temperature, affecting its conductivity and impedance. This incorporation also slightly increased the antimicrobial strength of chitosan due to the free drying of the composite solution without heating.

The antimicrobial findings exhibited moderate effectiveness against *Staphylococcus aureus, Candida albicans*, and *Aspergillus niger*, whereas no effectiveness was noted against *Escherichia coli*. The comparatively restricted antimicrobial efficacy in relation to CuO nanoparticle-based systems is ascribed to the lack of free CuO phases and the limited long-range ionic conduction within the composite. These results demonstrate a direct relationship among processing conditions, dielectric relaxation characteristics, and antimicrobial efficacy. The research emphasizes the significance of managing copper speciation and polymer–ion interactions when developing chitosan-based multifunctional materials intended for dielectric, biomedical, and antimicrobial uses.

## Figures and Tables

**Table 1:** Preparation of chitosan–copper (II) nitrate (Cs–Cu(NO_3_)_2_) films.

Copper nitrate concentration (wt%)	3 wt%	6 wt%	9 wt%
Amount of Cu(NO_3_)_2_ added	15 mg	30 mg	45 mg
Amount of Cu in Petri dish	5.082 mg	10.164 mg	15.246 mg
Amount of Cu in agar sample	12.74 µg	25.48 µg	38.22 µg
Approximate sample area	~2 cm^2^/(π × 25 cm^2^)		

**Table 2:** Scherrer crystallite size summary.

Sample	2θ (°)	FWHM (°)	D (nm)	Phase	Interpretation
S1 (0%)	10.0	2.5	31.9	Chitosan	Amorphous chitosan domain (broad hump)
S3 (3%)	22.6	1.5	54.0	Cu(NO_3_)_2_·3H_2_O	Emergent Cu-salt nanodomains at 3% loading
	26.4	1.0	81.6	Cu(NO_3_)_2_·3H_2_O	Larger domains; partial crystalline ordering
S6 (6%)	22.9	1.2	67.6	Cu(NO_3_)_2_·3H_2_O	Well-developed crystalline domains
	27.0	0.9	90.8	Cu(NO_3_)_2_·3H_2_O	Largest domain: optimal crystallization at 6%
S9 (9%)	27.6	1.1	74.4	Cu(NO_3_)_2_·3H_2_O	Domain size reduction vs. S6; matrix disruption
	29.0	1.0	82.1	Cu(NO_3_)_2_·3H_2_O	New reflection at 9%; phase heterogeneity

**Table 3:** XRD peak indexing data for S1–S9 series.

Sample	2θ (°)	d (Å)	FWHM (°)	D (nm)	Phase	Reflection/Assignment
S1 (0%)	10.0	8.84	2.5	31.9	Chitosan	(020) interchain spacing
	20.0	4.44	4.0	20.2	Chitosan	(110) intermolecular distance
S3 (3%)	10.5	8.42	2.0	39.9	Chitosan	(020) interchain spacing
	20.1	4.41	3.5	23.1	Chitosan	(110) intermolecular distance
	22.6	3.93	1.5	54.0	Cu(NO_3_)_2_·3H_2_O	Cu-salt emergence; weak crystallite nucleation
	26.4	3.37	1.0	81.6	Cu(NO_3_)_2_·3H_2_O	Crystalline Cu salt/overlap with chitosan
S6 (6%)	10.2	8.67	1.8	44.3	Chitosan	(020) interchain spacing
	22.9	3.88	1.2	67.6	Cu(NO_3_)_2_·3H_2_O	Well-defined Cu salt crystalline phase
	27.0	3.30	0.9	90.8	Cu(NO_3_)_2_·3H_2_O	Dominant crystalline peak; largest domain size
	32.0	2.79	1.0	82.6	Cu(NO_3_)_2_·3H_2_O/CuO	Mixed phase; trace CuO possible
S9 (9%)	10.1	8.75	2.2	36.3	Chitosan	(020) interchain spacing; slight broadening
	27.6	3.23	1.1	74.4	Cu(NO_3_)_2_·3H_2_O	Cu salt phase; smaller domain vs. S6
	29.0	3.08	1.0	82.1	Cu(NO_3_)_2_·3H_2_O	Additional Cu salt reflection at 9% loading

**Table 4:** Estimated crystallinity index (CI) of Cu(NO_3_)_2_ doped chitosan films.

Sample	Peak I (cts)	Avg BG I (cts)	CI (%)	Structural Comment
S1 (0%)	157	121	22.9	Semi-crystalline; typical of film-cast chitosan
S3 (3%)	242	179	26.0	Moderate increase; onset of Cu-salt ordering
S6 (6%)	273	196	28.2	Highest CI; Cu(NO_3_)_2_·3H_2_O crystalline micro-phase
S9 (9%)	172	133	22.7	CI drops back to S1 level; matrix disruption

**Table 5:** Lists the values of Eopt and n for the film samples.

Sample	Eopt	n
0	2.66	2.50
3	2.57	2.52
6	2.68	2.49
9	2.8	2.45

**Table 6:** The antimicrobial activity of chitosan and chitosan films with copper nitrate concentrations (0, 3, 6, and 9%) against different test microbes representing G+ve bacteria (*S. aureus*), G−ve bacterium (*E. coli*), Yeast (*C. albicans*), and fungi (*A. niger*).

Sample Name	Clear Zone (φmm)			
	*Staphylococcus aureus*	*Escherichia coli*	*Candida albicans*	*Aspergillus niger*
Cs	0	0	0	0
Cs-Cu(NO_3_)_2_ (3%)	0	0	15 ± 0.38 *	17 ± 0.46 *
Cs-Cu(NO_3_)_2_ (6%)	16 ± 0.38 *	0	17 ± 0.47 *	0
Cs-Cu(NO_3_)_2_ (9%)	19 ± 0.41 *	0	20 ± 0.36 *	18 ± 0.39 *

Results are expressed as mean ± standard deviations (SD) of five determinations. * highly significant.

## Data Availability

All data generated or analyzed during this study are included in this published article.
